# Lymphatic Filariasis: A Method to Identify Subclinical Lower Limb Change in PNG Adolescents

**DOI:** 10.1371/journal.pntd.0001242

**Published:** 2011-07-19

**Authors:** Susan Gordon, Wayne Melrose, Jeffrey Warner, Petra Buttner, Leigh Ward

**Affiliations:** 1 School of Public Health, Tropical Medicine and Rehabilitation Sciences, James Cook University, Townsville, Australia; 2 Microbiology and Immunology, School of Veterinary and Biomedical Sciences, James Cook University, Townsville, Australia; 3 School of Chemistry and Molecular Biosciences, University of Queensland, St Lucia, Brisbane, Australia; Centers for Disease Control and Prevention, United States of America

## Abstract

Lymphedema related to lymphatic filariasis (LF) is a disabling condition that commonly manifests in adolescence. Fifty-three adolescents, 25 LF infected and 28 LF non-infected, in age and sex-matched groups, using the Binax ICT rapid card test for filarial antigen were recruited to the study. None of the participants had overt signs of lymphedema. Lymphedema assessment measures were used to assess lower limb tissue compressibility (tonometry), limb circumference (tape measure), intra- and extra-cellular fluid distribution (bioimpedance) and joint range of motion (goniometry). The mean tonometric measurements from the left, right, and dominant posterior thighs were significantly larger in participants with LF compared to participants who had tested negative for LF (p = 0.005, p = 0.004, and p = 0.003, respectively) indicating increased tissue compressibility in those adolescents with LF. ROC curve analysis to define optimal cut-off of the tonometry measurements indicated that at 3.5, sensitivity of this potential screening test is 100% (95%-CI = 86.3%, 100%) and specificity is 21.4% (95%-CI = 8.3%, 41.0%). It is proposed that this cut-off can be used to indicate tissue change characteristic of LF in an at-risk population of PNG adolescents. Further longitudinal research is required to establish if all those with tissue change subsequently develop lymphedema. However, thigh tonometry to identify early tissue change in LF positive adolescents may enable early intervention to minimize progression of lymphedema and prioritization of limited resources to those at greatest risk of developing lifetime morbidity.

## Author Summary

The effects of lymphatic filariasis (LF) on the lymphatic system often become apparent during adolescence when the lower limb swells due to lymphedema and males develop hydrocele. Currently there is no simple or mobile field method to identify those at greatest risk of developing lymphedema or those with early subclinical lower limb change. Fifty-three adolescents, 25 LF infected and 28 LF non-infected were recruited to the study. The groups were compared with respect to lower limb tissue compressibility (tonometry), limb circumference (tape measure), intra- and extra-cellular fluid distribution (bioimpedance) and hip, knee and ankle joint range of motion (goniometry). Tonometry, is a simple, inexpensive tool, which measures the distance a plunger will indent the soft tissues. Those adolescents who were LF positive had significantly increased soft tissue compressibility when assessed with tonometry than adolescents who were LF negative. Tonometry has high levels of sensitivity to identify adolescents who test positive to LF. If we are able to identify adolescents before they have overt symptoms, management practices to decrease disease progression can be implemented. This could prevent lifetime morbidity and allow allocation of scarce resources to those identified to be most at risk of developing lymphedema.

## Introduction

The mosquito-borne parasitic disease lymphatic filariasis (LF) is endemic in around 81 tropical countries, has a global burden of around 120 million cases, and is classified by the World Health organization as the second most common cause of long term disability after mental illness [1]. Three species of filarial parasites cause LF. *Wuchereria bancrofti*, the cause of Bancroftian filariasis, accounts for 90% of the cases worldwide. Brugian filariasis is caused by *Brugia malayi*, which is found in eastern Asia, and *Brugia timori*, which is confined to Timor and adjacent islands. All three species cause similar lymphatic disease but only Bancroftian filariasis causes hydrocele and all are controlled and treated by the same methods [2].

LF has a wide clinical spectrum ranging from debilitating acute bacterial dermatolymphgangioadenitis (ADLA) attacks, covert lymphatic and renal disease, and various degrees of lymphedema, to the terrible disfiguring, and often socially ostracizing, chronic manifestations of hydrocele and elephantiasis [3].

A global program to eliminate LF as a public health problem was introduced in 2002 [1], [3]. It is based upon two “pillars.” Pillar one is the interruption of transmission by the use of community-wide preventative chemotherapy (PCT). This used to be called “mass drug administration”, MDA. The second pillar is the alleviation of suffering in those who already have chronic manifestations of the disease.

Over time filarial infection can lead to the development of chronic lymphedema. Infection often occurs in childhood but the obvious clinical effects of the disease such as filarial lymphedema or hydrocele may not occur until they reach adolescence [4]. Shenoy et al [5], [6] have shown covert abnormalities in the lymphatics with lymphoscintigraphy and worm nests by Doppler ultrasonography in *B.malayi* LF-infected adolescents as young as three years. Importantly, it has also been shown that these early lymphatic changes can be reversed by rug administration [7].

The mechanisms involved in the development of filarial lymphedema are not fully understood but they are known to be a complex interaction between the parasite and the host's immune system [8], [9].

Filarial lymphedema is painful and debilitating, accompanied by skin changes, decreased joint range of motion and recurrent infections. The presence of childhood filarial lymphedema results in social problems including embarrassment, frequent absence from school and even discontinuation of studies [10]. It is generally accepted that acute dermatolymphangioadenitisis the main risk factor for progression of lymphedema and incidence of these attacks can be greatly reduced by the introduction of a basic “self care” program consisting of daily limb washing, treatment of skin entry lesions, antibiotic treatment of established infections, gentle exercise, appropriate footwear and limb elevation [11]–[13].

Although lymphoscintigraphy is an effective method for detecting covert lymphatic changes, it is invasive in that it requires the injection of a radioactive substance and is not suitable for use in field-based research and the monitoring and evaluation, or “fine tuning” of treatment programs for morbidity control.

This study was undertaken to establish a non-invasive method to identify early, sub-clinical changes of lymphedema secondary to LF in the lower limbs of adolescents in Papua New Guinea (PNG). PNG was chosen because of the high Bancroftian filarial antigen prevalence. There is a national plan for the elimination of LF but PCT has only been started in two out of 19 provinces, and there is currently no morbidity control component in place [2].

## Methods

### Ethics statement

Ethics approval was obtained from the PNG Medical Research Advisory Council (MRAC number 08.25) and James Cook University Human Ethics Committee, Townsville, Australia. The proposed study was carefully explained to community leaders, school teachers, and health care workers in English, *Hiri Motu* and *Tok Pisin*. Parents provided verbal consent for their adolescents to participate. In this community of low literacy the use of verbal consent is appropriate and was approved by both the PNG Medical Research Advisory Committee and James Cook University Human Ethics Committee.

### Participants

The research was undertaken at Opau village, in central Province whilst a baseline survey for the PNG national LF program was conducted. Members of the village where inducted into the study based on their willingness to participate after the introduction (*tok save*). As adolescents (aged 10–21 years) were inducted, their LF status was determined using the Binax ICT rapid card test for filarial antigen [2] and demographic data was collected. These individuals were divided into LF reactive and LF non-reactive groups by the investigators responsible for sampling and testing. Binax ICT testing was conducted according to the manufacturers' instructions. An age and sex matched sub-sample of each group were selected for participation. Verbal consent was sought and obtained from the parents of those inducted. The researcher responsible for all measurements of the adolescents was blinded to the results of the ICT test.

There are many methods available to assess the lymphatic system. It was important that the methods included were inexpensive, portable and easy to apply in the field and therefore usable in the context of PNG. Hence the following methods were included in the study:

Circumferential measures of each lower limb were undertaken using a tape measure following the protocol described by the Australasian Lymphology Association [14]. This provided a gross measure of limb size. Measurements were performed at the metatarsophalangeal joints (MTP) (tape applied around the foot from MTP 1 to MTP5), the foot adjacent to the leg (tape applied around the dorsum and plantar aspect of the foot as close as possible to the leg), and 10 and 15 centimetres above and below the joint line of the knee. The distance from the knee was measured with a rigid ruler from the medial and lateral joint lines of the knee.

Tonometry was used to measure alterations in tissue resistance. The tonometer consists of a plunger device which, when applied to the skin, provides a measure of the ability of the skin and underlying tissue to resist compression. A higher tonometry value indicates greater indentation and decreased tissue resistance. It has been used to investigate post-surgical lymphedema [15] and LF in adults with established stage II and III lymphedema [16].

Tonometry was undertaken using a Flinders Tissue Tonometer (Flinders Medical Centre Biomedical Engineering, Australia) consisting of a central plunger operating through a 6 cm diameter footplate that rests on the skin and applies a load of 200 grams. The degree of penetration of the plunger is measured by a micrometer on a linear scale [15]. The tonometer was calibrated according to the manufacturers' instructions prior to each measurement session. The length of the posterior thigh was measured from the gluteal fold to the posterior knee crease using a tape measure. The value was halved and this mid-point in the centre of the posterior thigh was marked. The same distance from the superior aspect of the patella was marked on the anterior thigh. The length of the calf was measured from the posterior knee crease to the base of the heel. This value was halved and a point marked on the centre of the posterior calf. The tonometer was placed on the skin at each marked point and readings to the nearest millimeter were recorded.

Goniometry was used to measure joint range of motion (ROM). When lymphedema is present and limb size increases the range of movement at joints can become restricted. Goniometry was undertaken for ankle dorsiflexion, knee flexion and hip flexion of each lower limb [17].

Bioimpedance spectroscopy (BIS) measures the opposition or impedance (Z) to the flow of an alternating electrical current passed through the body. The SFB7 used in this study performs measurements at 256 frequencies over the range 3–1000 KHz.. At low frequencies, <20 kHz, the current passes predominantly through the extra-cellular fluid (ECF) while at a high frequencies, >50–100 kHz, it passes through both the intra- cellular fluid (ICF) and the ECF. Thus the ratio of impedances, measured at high and low frequencies, provides an indication of change in fluid distribution between the tissue compartments. In secondary lymphedema, increased volumes of ECF are expected and hence the ratio of ICF to ECF should decrease. Since impedance is inversely related to fluid volume this would be observed as an increase in the impedance ratio Ri∶Re (intracellular impedance: extracellular impedance^1^).

Evidence exists that bioimpedance spectroscopy can identify a change in fluid distribution in the arms of women at risk of lymphedema following management for breast cancer up to six months earlier than the usual clinical method of circumferential measurement [18] . Bioimpedance instruments are light and portable and may provide a more accessible and reasonable method to identify early accumulation of fluid in the limbs of adolescents.

Tissue bioimpedance was measured for each lower limb using an Impedimed SFB7 bioimpedance spectrometer device (ImpediMed Limited, Unit 1–50 Parker Court, Pinkenba, Qld, 4008 Australia). Adhesive electrodes were attached to the feet and hands of participants and the impedance recorded according to the manufacturer's instructions. All data was collected between the hours of 11am and 3pm over three consecutive days. All measurements were made with the subjects in the same position, supine. There is no reason to anticipate that BIS will perform any differently in a rural/village environment to elsewhere assuming a consistent procedure is used. However, as no reliability studies regarding the use of this equipment in a field environment were available two bioimpedance measures were undertaken for each limb. Bioimpedance values, goniometry and circumferential measures are altered by limb composition hence participants were asked which leg they would kick a ball with to determine limb dominance.

### Statistical analysis

Numerical variables were described using mean values and standard deviations (SD) when found to be approximately normally distributed. Median values and inter-quartile ranges (IQR) were used when the variable was skewed. Participants with and without lymphatic filariasis (LF) were compared using t-tests, Fisher's exact tests, and non-parametric Wilcoxon Mann-Whitney test. Binary logistic regression analyses were conducted to identify associations between lower limb measurements and LF. Demographic and body characteristics were considered as potential confounders. The model was adjusted for a confounder when the estimate changed by about 10% or more. Paired analyses comparing left and right limb were conducted using paired t-tests and paired non-parametric Wilcoxon signed rank tests. Throughout the analysis a significance level of 0.05 was assumed. Statistical analysis was conducted using PASW (version 18 of SPSS; SPSS Inc. IBM; Chicago; Illinois).

## Results

**Figure 1:**
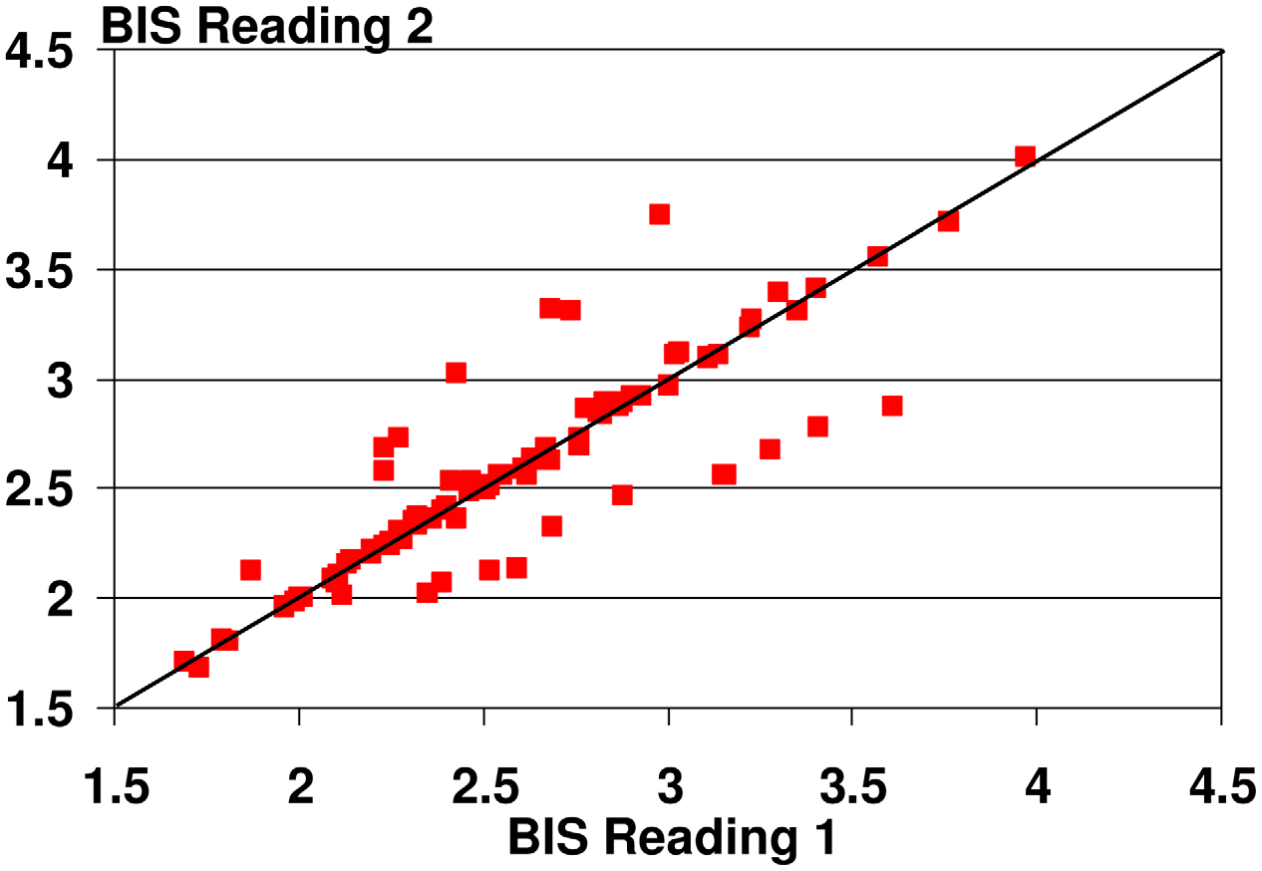
Concordance correlation coefficient for repeated BIS measures.

In order to assess the reliability of the BIS measures we calculated the concordance correlation coefficient by Lawrence I-Kuei Lin [19]. The concordance correlation coefficient was high with 0.88 (95%-confidence interval 0.82, 0.92) suggesting good reliability of the measurement (Figure 1).

Mean age of the 53 participants was 16.5 years (SD 2.5; range 10 to 21 years) and 54.7% were female. Overall 47.2% tested positive for LF. The mean weight of the participants was 50.6 kg (SD 7.1) and the mean body mass index (BMI) was 19.7 kg/m^2^ (SD 1.9; range 14.5 to 23.6). Four participants (7.5%) were left leg dominant and one participant (1.9%) would use both legs equally to kick a ball (Table 1). None of the demographic and body characteristics were significantly different for participants with and without LF (Table 1).

**Table 1:** Demographics and body characteristics of participants stratified and compared by Lymphatic Filariasis (LF) status.

Characteristic	Total n = 53	LF negative n = 28	LF positive n = 25	p-value
Mean age (SD)* [years]	16.5 (2.5)	16.0 (2.6)	17.0 (2.3)	P = 0.163
% Female	54.7%	46.4%	64.0%	P = 0.271
Mean height (SD) [cm]	159.8 (6.7)	160 (7.1)	159.6 (6.3)	P = 0.822
Mean weight (SD) [kg]	50.6 (7.1)	49.6 (6.1)	51.7 (8.0)	P = 0.293
Mean BMI** (SD) [kg/m^2^]	19.7 (1.9)	19.3 (1.6)	20.2 (2.2)	P = 0.111
% Dominant leg left or both	9.4%	7.1%	12.0%	P = 0.658

*SD = standard deviation;

**BMI = body mass index.

The mean tonometric measurements from the left, right, and dominant posterior thighs were significantly higher in participants with LF compared to participants who had tested negative for LF (p = 0.005, p = 0.004, and p = 0.003, respectively; Table 2). Logistic regression showed increasing the tonometric measurement from the dominant posterior thigh by 1 unit increased the odds of having tissue change secondary to LF by 2.2 (95%-CI = (1.2, 4.1); p = 0.014). This result was adjusted for the confounding effects of BMI.

**Table 2:** Bioimpedance, range of motion, circumferential and tonometry measures for all participants and stratified for LF status.

Characteristic	Total n = 53	LF negative n = 28	LF positive n = 25	p-value
**Tonometry (mm)**				
L mid-calf; mean (SD)	4.03 (0.90)	3.97 (0.99)	4.10 (0.81)	P = 0.589
R mid-calf; mean (SD)	3.82 (0.81)	3.89 (0.74)	3.74 (0.89)	P = 0.496
D mid-calf; mean (SD)	3.86 (0.78)	3.87 (0.71)	3.86 (0.86)	P = 0.979
L posterior thigh; mean (SD)	4.59 (1.20)	4.17 (1.11)	5.07 (1.13)	P = 0.005#
R posterior thigh; mean (SD)	4.80 (1.13)	4.39 (1.08)	5.26 (1.03)	P = 0.004#
D posterior thigh; mean (SD)	4.80 (1.11)	4.38 (1.07)	5.26 (0.99)	P = 0.003#
L anterior thigh; mean (SD)	5.31 (1.03)	5.13 (1.0)	5.5 (1.0)	P = 0.190
R anterior thigh; median (IQR)**	5.43 (4.51, 5.96)	5.36 (4.35, 5.92)	5.52 (4.81, 6.05)	P = 0.387
D anterior thigh; median (IQR)	5.45 (4.53, 6.00)	5.38 (4.37, 5.92)	5.60 (4.81, 6.05)	P = 0.331
**BIS ratio (Ri∶Re)**				
R lower limb; mean (SD)*	2.58 (0.47)	2.56 (0.54)	2.59 (0.38)	P = 0.798
L lower limb; mean (SD)	2.62 (0.47)	2.64 (0.51)	2.59 (0.43)	P = 0.703
D lower limb; mean (SD)	2.57 (0.47)	2.55 (0.54)	2.59 (0.38)	P = 0.743
**Goniometry (degrees)**				
R ankle dorsi-flexion ROM; mean (SD)	7.5 (3.0)	7.5 (2.7)	7.4 (3.5)	P = 0.944
L ankle dorsi-flexion ROM; mean (SD)	7.2 (3.3)	7.0 (3.0)	7.3 (3.7)	P = 0.731
D ankle dorsi-flexion ROM; mean (SD)	7.4 (3.2)	7.6 (2.7)	7.3 (3.6)	P = 0.791
R knee flexion ROM; mean (SD)	144.7 (4.1)	144.4 (5.1)	145.0 (2.7)	P = 0.570
L knee flexion ROM; mean (SD)	144.8 (6.8)	144.8 (6.3)	144.8 (7.4)	P = 0.992
D knee flexion ROM; mean (SD)	144.7 (3.9)	144.4 (4.8)	145.0 (2.8)	P = 0.630
R hip flexion ROM; mean (SD)	103.9 (8.1)	104.8 (9.3)	103.0 (6.7)	P = 0.417
L hip flexion ROM; mean (SD)	104.6 (7.9)	104.3 (7.2)	104.9 (8.7)	P = 0.785
D hip flexion ROM; mean (SD)	104.2 (8.0)	104.9 (9.2)	103.3 (6.5)	P = 0.470
**Circumferential measures (cm)**				
L MTP; mean (SD)	23.0 (1.5)	23.1 (1.5)	22.9 (1.5)	P = 0.606
R MTP; mean (SD)	23.0 (1.5)	23.0 (1.3)	22.9 (1.6)	P = 0.940
D MTP; mean (SD)	23.0 (1.4)	23.0 (1.3)	22.9 (1.6)	P = 0.877
L dorsum; mean (SD)	24.8 (1.8)	25.0 (2.0)	24.5 (1.6)	P = 0.394
R dorsum; mean (SD)	24.6 (1.6)	24.5 (1.5)	24.6 (1.6)	P = 0.837
D dorsum; mean (SD)	24.6 (1.5)	24.6 (1.5)	24.6 (1.6)	P = 0.904
L 10 cm proximal; mean (SD)	36.7 (2.8)	36.0 (2.0)	37.4 (3.4)	P = 0.063
R 10 cm proximal; mean (SD)	36.9 (2.9)	36.1 (2.0)	37.8 (3.4)	P = 0.038#
D 10 cm proximal; mean (SD)	36.9 (2.9)	36.1 (2.0)	37.7 (3.4)	P = 0.042#
L 10 cm distal; mean (SD)	30.8 (1.9)	30.5 (1.5)	31.2 (2.3)	P = 0.172
R 10 cm distal; mean (SD)	30.8 (2.0)	30.5 (1.5)	31.2 (2.4)	P = 0.174
D 10 cm distal; mean (SD)	30.8 (2.0)	30.5 (1.5)	31.2 (2.4)	P = 0.180
L 15 cm proximal; mean (SD)	40.8 (3.1)	40.0 (2.5)	41.7 (3.5)	P = 0.043#
R 15 cm proximal; mean (SD)	40.8 (4.0)	40.4 (2.3)	41.3 (5.4)	P = 0.410
D15 cm proximal; mean (SD)	40.8 (4.0)	40.4 (2.3)	41.3 (5.3)	P = 0.445
L 15 cm distal; mean (SD)	30.8 (3.0)	31.1 (3.1)	30.5 (2.9)	P = 0.462
R 15 cm distal; mean (SD)	30.9 (2.6)	30.8 (2.7)	31.0 (2.6)	P = 0.722
D 15 cm distal; mean (SD)	30.9 (2.6)	30.8 (2.7)	31.0 (2.6)	P = 0.786

*SD = standard deviation;

**IQR = inter-quartile range;

# = significant p-value.

right limb (R), left limb (L) and dominant limb (D).

The mean circumferential measurements of the right and dominant thighs, 10 cm proximal to the knee, and of the left thigh, 15 cm proximal to the knee were significantly greater in participants with LF compared to participants who had tested negative for LF (p = 0.038, p = 0.042 and p = 0.043, respectively; Table 2). However when adjusted for the confounding effects of BMI those measurements were no longer significantly associated with LF (p = 0.164, p = 0.189, p = 0.226, respectively).

There were no significant differences found in the impedance ratio of the legs between LF positive and LF negative subjects, irrespective of which leg was compared or of limb dominance (Table 2). However there was a significant difference in the bioimpedance ratios of the dominant and non-dominant legs of the LF negative group. This relationship was not identified in the LF positive group (Table 3) possibly suggesting that BIS is detecting an LF-related change in ECW∶ICW ratios but that this is confounded by limb dominance effects upon the impedance measurements.

**Table 3:** Bioimpedance, range of motion, circumferential and tonometry measures comparing dominant with non-dominant limbs.

Characteristic	Total n = 53	LF negative n = 28	LF positive n = 25
**BIS ratio (Ri∶Re)**			
Lower limb; mean (SD)*	P = 0.112	P = 0.003#	P = 0.935
**Goniometry (degrees)**			
Ankle dorsi-flexion; mean (SD)	P = 0.462	P = 0.159	P = 0.841
Knee flexion; mean (SD)	P = 0.869	P = 0.740	P = 0.981
Hip flexion; mean (SD)	P = 0.928	P = 0.532	P = 0.404
**Circumferential measures (cm)**			
MTP; mean (SD)	P = 0.518	P = 0.512	P = 0.899
Dorsum; mean (SD)	P = 0.362	P = 0.285	P = 0.720
10 cm proximal; mean (SD)	P = 0.132	P = 0.342	P = 0.250
10 cm distal; mean (SD)	P = 0.851	P = 0.926	P = 0.862
15 cm proximal; mean (SD)	P = 0.983	P = 0.231	P = 0.577
15 cm distal; mean (SD)	P = 0.826	P = 0.666	P = 0.282
**Tonometry (mm)**			
Mid-calf; mean (SD)	P = 0.277	P = 0.396	P = 0.501
Posterior thigh; mean (SD)	P = 0.132	P = 0.265	P = 0.325
Anterior thigh; mean (SD)	P = 0.426	P = 0.829	P = 0.367

*SD = standard deviation;

# = significant p-value.

These results support the use of tonometry of the dominant posterior thigh to indentify alteration in tissue compressibility in adolescents with sub-clinical lymphedema secondary to LF. Mean and standard deviation values for dominant posterior thigh tonometry in the positive LF group were 5.26 and 0.99 and in the negative LF group were 4.38 and 1.07. Receiver operating characteristics (ROC) curves were plotted to identify the optimal cut-off for these measurements to differentiate between those with and those without altered tissue compressibility related to LF. Power when comparing tonometry of the dominant posterior thigh in adolescents who are LF positive and LF negative was 86.3%.

**Figure 2:**
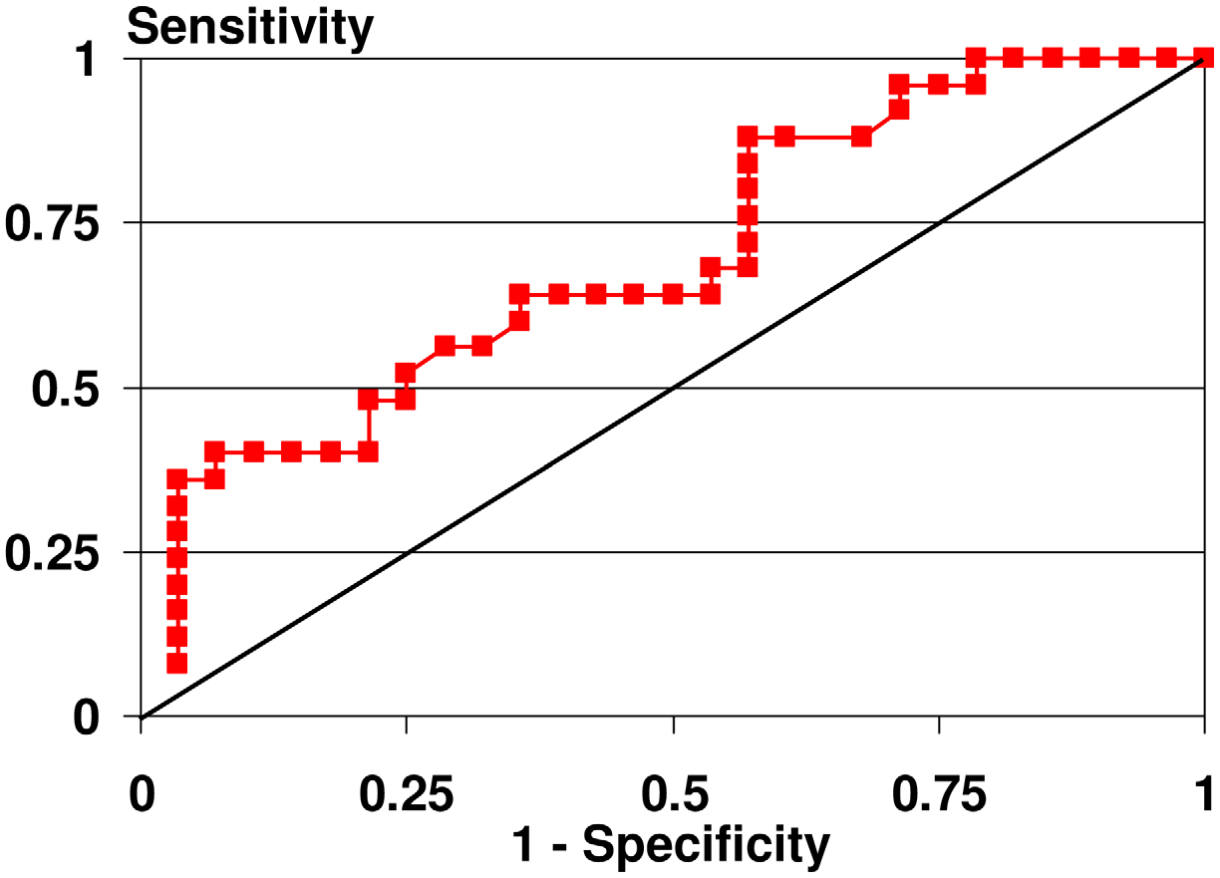
Receiver Operating Characteristic (ROC) curve for tonometry measurement of tissue compressibility of the dominant posterior thigh.

ROC curve analysis to define optimal cut-off of the tonometry measurements of the dominant posterior thigh to identify early tissue changes is reported in Figure 2 and Table 4.

**Table 4:** Lymphatic Filariasis classification using the suggested tonometric dominant thigh measurement as a screening test.

	Disease status		
Tonometry	LF positive	LF negative	Total
**Positive**	25 (100%)	22	47
**Negative**	0	6 (21.4%)	6
**Total**	25	28	53

If we chose as the cut-off the 3.5, then sensitivity of this potential screening test is 100% (95%-CI = 86.3%, 100%) and specificity is 21.4% (95%-CI = 8.3%, 41.0%). All true LF cases are detected as such, but together with 22 false positive cases. The positive predictive value is 53.2%. However the false positive cases could be picked up in a second test.

## Discussion

Digital tonometry identified increased tissue compressibility in the posterior thigh in LF-infected adolescents with no overt signs of secondary lymphedema. As there was no significant concomitant change in BIS values these findings indicate a softening of tissue rather than altered intra- and extra-cellular fluid distribution as the first measurable LF tissue changes. This is in contrast to women who develop secondary lymphedema after breast cancer (BC) where alteration of extra- and intra-cellular fluid distribution is the earliest identifier of lymphatic change [18]. The difference in sensitivity of measurement tools in the LF and BC groups is likely to be related to the cause of lymphatic dysfunction. During intervention for cancer the removal of lymph nodes and direct trauma to the lymphatic tissue (surgical, radiation) are the main contributors to secondary lymphedema. In LF, dilation of the lymphatic vessels in response to the presence of the worm and the host inflammatory response to the living worm and their secreted antigens cause lymphatic damage [20]. Dilation of the lymphatics in this early stage is unlikely to lead to the accumulation of lymph seen in BC-related lymphedema and detected by BIS. Further, when the worm dies symbiotic Wolbachia organisms are released and introduce Wolbachia bacteria to the host which causes an inflammatory response in the lymphatic system [8]. It has recently been reported that living worms may also release bacteria and/or the products of the symbiotic Wolbachia into their host [21]. A study of Rhesus monkeys identified that they mount an antibody response to Wolbachia surface proteins that are temporarily associated with worm death and lymphedema development [8]. Specifically, increased Th1/Th17 responses and decreased regulatory T cells as well as regulation of Toll- and Nod-like receptors have been identified in the pathogenesis of filarial lymphedema [20]. Intra-subject between limb variation in BIS is expected due to limb dominance and consequent variation in limb mass and composition. This was not identified in the LF positive group.

Research is required to determine if increased tissue compressibility is due to fatty infiltration, collagen breakdown, loosening of inter-cellular junctions or simply dilation of lymph vessels.

Two sets of genetic risk factors have been suggested for the development of lymphedema, immune response genes associated with heightened inflammatory responses and lymphatic damage following filarial infection and genes associated with impaired lymphangiogenesis [8].

Thigh measures are likely to show the earliest change due to the heavy worm burden in the groin associated with LF. In adult males the worms have been identified by ultrasound to have a preference for the vessels associated with the spermatic cord [22] while in young boys they are found in the inguinal or other peripheral lymph nodes [8] and in the scrotal lymphatic vessels [23]. It has been suggested therefore that puberty and hormonal change may alter worm infestation. This may have contributed to why BIS did not detect differences in this study. The electrode placement in this study measured the impedance of the whole lower limb. As impedance is inversely proportional to the cross sectional area of the limb this results in the impedance of the whole leg being dominated by that of the smaller cross-sectional area of the lower leg, calf and below. Therefore even if impedance of the thigh is different the sensitivity to detect this difference is decreased when measuring the whole leg. BIS sensitivity should be improved by altering electrode placement and measuring the thigh region only.

Tonometry is simple to learn, non-invasive, portable, does not require electricity or batteries and is relatively inexpensive (<$500AUD). After a short training session (30 minutes), subsequent practice and establishment of intra-measurer reliability it would be suitable for use by community health workers. The training session should include the manufacturers requirements for calibration of the tonometer on a flat surface prior to measurement, placement of the tonometer in contact with the skin surface to be measured in a vertical position and reading of the measurement dial. As each measurement takes less than a minute one tonometer would allow a large number of people to be assessed. Tonometry could be used to screen for onset of tissue change, to monitor tissue change and the effect of interventions in those with established lymphedema. A digital tonometer is being developed that may allow even easier field use.

Tonometry is most reliable when assessed on an even surface. This may account for the difference in findings for the posterior and anterior thigh tonometry measures as when lying in the prone position the posterior thigh provides a more even surface than the anterior thigh in the supine position. There are many factors which contribute to the development of lymphatic change in the LF population, many of which are not well understood. It is not known if all the positive LF adolescents identified in this study will undergo progressive lymphatic change.. Further research is required to track changes in tonometry, circumferential measures and BIS in these participants and better understand the progression of lymphatic and tissue change related to LF.

This study suggests possible cut-off values for tonometry which may indicate tissue change characteristic of LF in an at-risk population of PNG adolescents. However, this should not be generalized to other populations and requires further research to establish its generalisability even within PNG. The cut-off chosen in this analysis results in low specificity and a high number of false positives. However the consequences of being wrongly treated are little in comparison to unidentified and unmanaged lymphedema.
